# Gender-based violence against women during the COVID-19 pandemic: recommendations for future

**DOI:** 10.1186/s12905-023-02372-6

**Published:** 2023-05-03

**Authors:** Abbas Ostadtaghizadeh, Mozhdeh Zarei, Nadia Saniee, Mohammad Aziz Rasouli

**Affiliations:** 1 Department of Health in Emergencies and Disasters, School of Public Health, Tehran University of Medical Sciences, Tehran, Iran; 2 Assistant Professor of Medical Library and Information Science, Department of Public Health, Asadabad School of Medical Sciences, Asadabad, Iran; 3 Department of Epidemiology and Biostatistics, Faculty of Medicine, Kurdistan University of Medical Sciences, Sanandaj, Iran

**Keywords:** Gender-based violence, Women, Scoping review, COVID-19, Health policy

## Abstract

**Background:**

Gender-based violence (GBV) includes any physical, sexual, psychological, economic harms, and any suffering of women in the form of limiting their freedom in personal or social life. As a global crisis, COVID-19 has exposed women to more violence, which requires serious actions. This work aims to review the most critical dimensions of the GBV against women, effective factors on it, and strategies for combating it during the COVID-19 pandemic in order to provide recommendations for future pandemics.

**Methods:**

This study was conducted based on PRISMA-ScR. First, PubMed, Embase, Scopus, Web of Science, ProQuest, and Google Scholar were searched in April 2021 with no time limitation and location using the related keywords to COVID-19 and GBV. The searched keywords were COVID-19, gender-based violence, domestic violence, sexual violence, women, violence, abuse, and their synonyms in MESH and EMTREE. Duplicates were removed, titles and abstracts were screened, and then the characteristics and main results of included studies were recorded in the data collection form in terms of thematic content analysis.

**Results:**

A total of 6255 records were identified, of which 3433 were duplicates. Based on inclusion criteria 2822 titles and abstracts were screened. Finally, 14 studies were eligible for inclusion in this study. Most of these studies were conducted in the United States, the Netherlands, and Iran, mostly with interventional and qualitative methods.

**Conclusions:**

Strengthening ICT infrastructure, providing comprehensive government policies and planning, government economic support, social support by national and international organizations should be considered by countries worldwide. It is suggested that countries provide sufficient ICT infrastructure, comprehensive policies and planning, economic support, social support by collaboration between national and international organizations, and healthcare supporting to manage incidence of GBV against women in future pandemics.

**Supplementary Information:**

The online version contains supplementary material available at 10.1186/s12905-023-02372-6.

## Introduction

International definitions of gender-based violence(GBV) and violence against women have emerged since the early 1990s [1]. GBV is a phenomenon deeply rooted in gender inequality, and continues to be one of the most notable human rights violations within all societies [2]. GBV as a main violence against women includes any physical, sexual, psychological, economic, and also any suffering of women in the form of restricting their freedom in personal or social life [3]. Most sexual violence is related to interpersonal relationships includes domestic violence, sexual violence, forced marriage, female genital mutilation, harassment, violence and abuse, and human trafficking [3, 4].

As an example of the impact of GBV on women, the results of studies from 2000 to 2018 showed that more than one in four women (27%) between the ages of 15 and 49 had ever have had a sexual partner, experienced physical or sexual violence, or both, since the age of 15 [5, 6].

In the past, crises have been associated with increased cases of GBV in natural disasters, including the earthquake in Haiti in 2007, Hurricane Katrina in 2005, and the eruption of Mount St. Helens in the 1980s due to unemployment, family, and other stressors has been reported [7–10]. According to researchers, epidemics cannot be excluded from this [11]. Recent outbreaks such as Ebola, Cholera, Zika, and Nipah have also led to an increase in cases of domestic violence [12]. Also, cases of sexual assault, violence against women, and rape also increased during the Ebola outbreak in West Africa [13].

GBV, already a global crisis before the pandemic, has intensified since the outbreak of COVID-19. Lockdowns and other mobility restrictions have left many women trapped with their abusers, isolated from social contact and support networks [14].

Health guidelines on quarantine and “stay home” during COVID-19 pandemic expose women to further damages. In this situation, many countries around the world, such as the United States, Ireland, China, the United Kingdom, and African have experienced a dramatic increase in domestic violence, which is one of the dimensions of GBV [4]. The results of studies have shown that China has witnessed a three-fold increase in domestic violence cases after the imposition of quarantine, and an increase of 21 to 35% in domestic violence was also reported in different states of the United States [15].

In the absence of a vaccine or effective treatment for Covid-19, quarantine for various periods of time has been used as an option by most countries, leading to lifestyle changes [16]. Most of the work is done from home and efforts are made to maintain social distance. These measures are critical to protecting health care systems [17]. However, positive efforts to combat COVID-19 have negative consequences associated with them. These negative consequences include the risk of job loss, economic vulnerabilities, and mental health issues due to isolation, loneliness, and uncertainty [16, 17].

Considering the importance of maintaining the safety and health of women as half of a society and their key roles in the family, especially during pandemics and crises, and looking at existing studies shows that different research have been carried out by one of the methods of literature review regarding one aspect of GBV against women. Organizations, researchers and civil society representatives have warned of an increase in reports of GBV against women during the Covid-19 pandemic. Concerns about this issue have been expressed through official and unofficial networks, and they have emphasized the need to create effective interventions to prevent and combat this phenomenon. The urgency of this situation requires an analysis of the available scientific literature on strategies and recommendations to deal with GBV against women in the context of social distancing measures adopted as a response to the COVID-19 pandemic.

## Methods

This scoping review is directly aligned with the Preferred Reporting Items for Systematic Reviews and Meta-Analyses extension for Scoping Reviews (PRISMA-ScR) Check- list [18]. The present study seeks to answer the following questions:

1. What were the most important dimensions of the gender-based violence against women during the COVID-19 pandemic?
2. What were the effective factors on increasing the gender-based violence against women during the COVID-19 pandemic?
3. What were the most important strategies to combat the gender-based violence against women during the COVID-19 pandemic?
4. Which strategies are recommended to manage the gender-based violence in future pandemics?

### Protocol and registration

We utilized the scoping review framework by Arksey and O’Malley (2005), as well as recent guidance to increase rigor and reporting of scoping reviews [19]. The a priori protocol for this review was drafted using the PRISMA extension for Scoping Reviews [18]. Due to the rapid nature of this review, the protocol for this review was not published, but can be accessed by contacting the authors.

### Eligibility criteria

The inclusion criteria were: Original articles, narrative reviews, short communications, and grey literature related to the GBV against women during the COVID-19 pandemic; Availability of Full-text; Published in the English.

The exclusion criteria were: Case studies, notes, letter to the editor, editorials, comments, conference papers, perspectives, systematic reviews, meta-analysis; and scoping reviews: studies related to domestic violence and sexual violence alone; studies focusing on the GBV against children and men.

### Information sources

We searched PubMed, Embase, Scopus, Web of Science, Proquest databases, and Google Scholar in April 2021 without time limitation and locations. The searched keywords were COVID-19, gender-based violence, domestic violence, sexual violence, women, violence, abuse, and their synonyms in MESH and EMTREE. The search strategies were provided by NS and approved by MZ. The search strategy for each database is mentioned in Appendix 1. The references of included documents were also reviewed to identify more related articles.

### Selection of sources of evidence

All searched records were imported into EndNote-8. After removing duplicates, the title and abstract of studies were screened. Finally, related full-texts were selected and then confirmed them. The most important reason for removing studies in the screening phase was focusing on other kinds of violence than GBV.

### Data extraction

A data extraction form was developed using Excel spreadsheet and bibliographic characteristics of each document including the first author, year of the research, research method, dimensions of the GBV against women, effective factors on it, and strategies for preventing it were recorded by MZ and approved by NS.

### Synthesis of results

The data analysis steps included familiarizing with the concept, determining primary codes, searching for semantic units in the text, reviewing semantic units, defining and naming semantic units, and reporting [20]. First, the gender-based violence was considered as a main theme. Second, dimensions of the GBV, effective factors on it, and key strategies for combating it during the COVID-19 pandemic were determined as sub-themes, then overlapping themes were merged together. Finally, narrative methods were applied to synthesize the extracted results and main strategies were recommended for future pandemics.

## Results

**Fig. 1:**
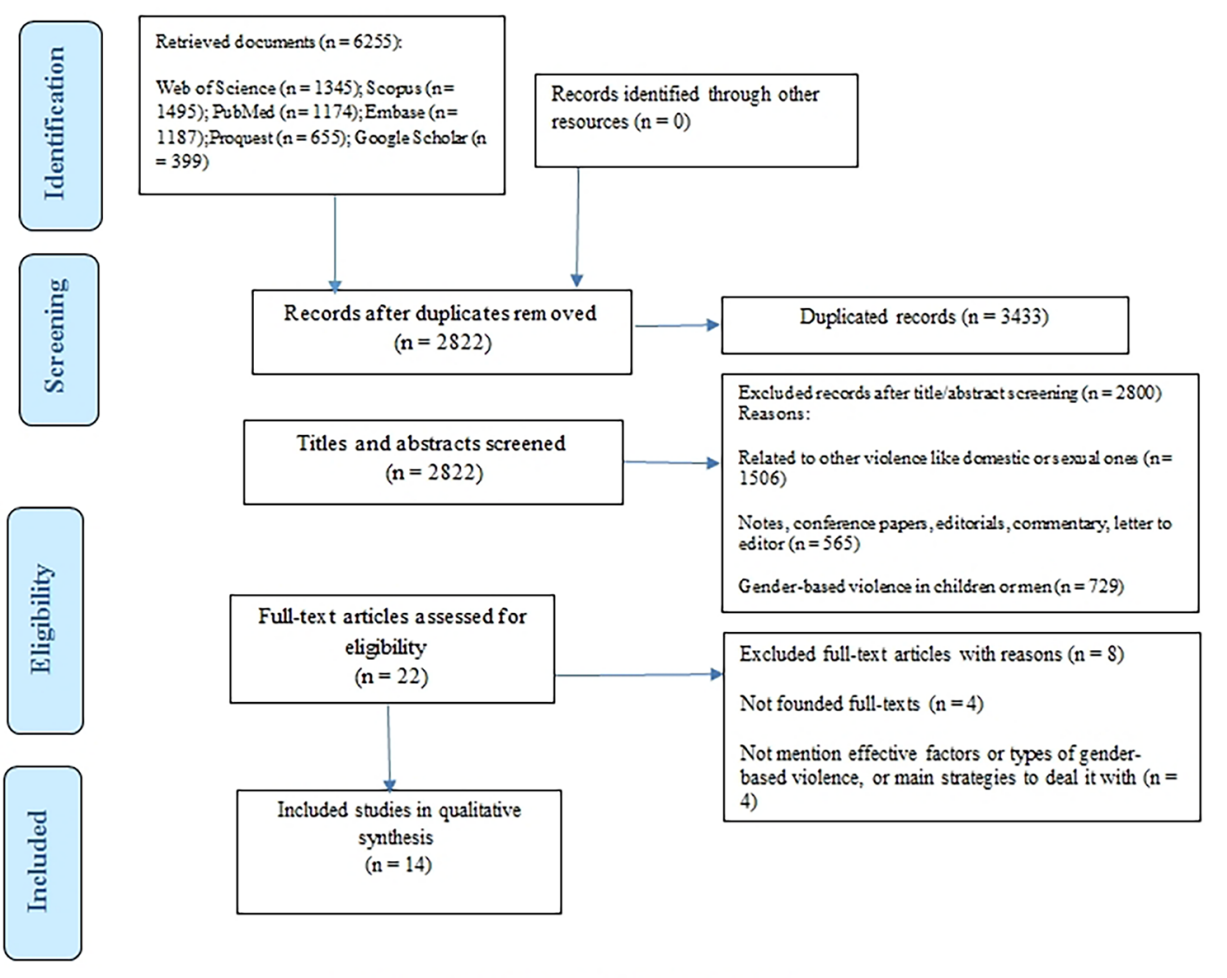
PRISMA diagram of search and selection process

After performing the search, a total of 6,255 records was identified, of which 3,433 were duplicates. In terms of inclusion criteria, 2822 titles and abstracts were screened. After studying 22 full texts, 14 ones were eligible for including in the study (Fig. 1).

### Quality assessment

The quality assessment of studies was not done due to the different methodology of the included studies and the type of review (Scoping reviews) [21].

### Measure characteristics

The bibliographic characteristics of these studies are presented in Table 1. The results showed that Turkey, the United Kingdom, the United States, and South Africa had the highest number of studies with two articles, and Indonesia, Italy, Nigeria, Colombia, Ecuador, and Brazil had the lowest number of studies with one study. Eight of these studies were conducted in the form of literature review and analysis of government documents, while two studies were survey, two studies were qualitative, one was observational, and one was a mixed methods study. A total of 12 studies were conducted in 2020 and two studies in 2021.

**Table 1:** Descriptive characteristics of selected studies

First author (year)	Country	Research Method	Types of gender-based violence	Factors affecting gender-based violence against women	Strategies to combat gender-based violence against women
**Yenilmez (2020)** [3]	Turkey	literature review	Sexual and physical	- Home quarantine and social distancing - Lack of responsive services - Work in the private and informal sectors - social norms - Lack of case officers - Lack of development plans - Lack of adequate shelter for victims - Inequality and economic recession - Sources of violence related to the virus - Consumption of drugs and alcohol by spouses and sexual partners - Lack of access to Internet communication networks - Not paying attention to the report of real statistics of violence against women - Age - Low level of education	- Providing guidelines and policies to combat gender-based violence - Providing welfare and support facilities and services (shelter, care, and online counseling services, women’s advocacy groups) - Training and equipping health workers - Educate people in the community about the effects of gender-based violence and help victims - Amend government laws to protect the foundation of the family and reduce the factors that aggravate domestic violence - Government financial investment to support women.
**Yenilmez (2020)** [4]	Turkey	literature review	Sexual, physical, psychological	- Quarantine and social distancing - Family economic problems - Psychological stress caused by a pandemic - Lack of men`s cooperation in family affairs - Lack of access to support services	- Women’s participation in charity and earning money - Establishment of shelters and centers for women protection - Set clear rules to protect women in pandemic situations - Creating mobile-based educational and communication programs - Existence of support systems at the micro level.
Polischuk (2020) [14]	USA	Observational	Physical, psychological, financial	-Quarantine and social distancing	- Establish laws to combat GBV - Creating communication lines and using social networks - Cooperation between governmental and non-governmental organizations - Provide instructions to divide household duties, food and social security, especially for bisexual people - Access to human and financial resources - Creating campaigns against GBV
Magezi (2020) [15]	South Africa	literature review	Physical, psychological, economic, sexual, femicide, or suicide	- Quarantine and its stress - Negative social norms and gender inequality - Country weak economy and no income spouse -Economic dependence on the spouse - Alcohol consumption at home - Lack of access to social and psychological support from the family, friends, and the community - The existence of an before abusive relationship - Dependence on children - Threat to death of wife or children	- Providing spiritual support from the church
Lund (2020) [7]	Brazil	literature review	Sexual, physical, psychological, economic	- Quarantine and social distancing - Lack of access to shelters - Lack of a system for registering gender-based violence cases	- Investment in online support services and public alarm systems - Providing telephone support lines - Cooperation between health organizations and non-governmental organizations which defend women - Financial and shelter support for women, especially refugees - Emergency accommodation for homeless women - Providing healthcare recommendations during the COVID-19 pandemic in the local languages - Providing individual sexual, physical, and psychological care services - Training psychologists to provide basic services to victims
Masigo (2020) [16]	South Africa	literature review	Physical, emotional, sexual violence, femicide	- Quarantine and social distancing - No income - Not dividing the household duties	- Providing social services
Londono (2021) [17]	Columbia	Mixed-methods	- Sexual, physical, mental, death, emotional, economic, inheritance, digital - Criminal violence and drug use, - Structural violence (government and society), - Direct violence (by family)	- Inequality in family responsibilities - Unemployment and financial dependence on the spouse - Horizontal inequality - The impact of government and social laws - Lack of accurate reporting of violence against women - The perception of violence against women as hallmark by the family and society	- Reforming government and social laws for women - Family-based social support networks - Creating an appropriate reporting system - Women’s protection movements
Speed (2020) [2]	the UK	Survey	Physical, psychological, sexual, and economic violence	- Quarantine and social distancing - Patriarchy and socialization processes - Consumption of alcohol and drugs - Inability to provide family expenses - Women`s isolation - No basic regulations - Disruption of social networks - Disruption of programs and implementation of laws and social support - Economic disorder - Failure to report cases of violence due to the lack of a transparent law - Closing refugees	- Establishment of social protection services for women who are under gender violence - Applying a comprehensive and inter-organizational approach - Performing activities to prevent gender-based violence - Prevent the entry of more refugees - Strengthening remote support infrastructure (web chat) - Government financial support for GBV organizations - Establishment of remote court services - Recording the statements of victims and witnesses electronically by the police - Community-based services - Financial support for the provision of remote and online services by sponsoring organizations and courts
**Solorzano (2020)** [5]	Ecuador	literature review	Physical, sexual, verbal, psychological, femicide	- Quarantine	-
Lund (2020) [7]	Brazil	literature review	Sexual, physical, psychological, economic	- Quarantine and social distancing - Lack of access to shelters - Lack of a system for registering gender-based violence cases	- Investment in online support services and public alarm systems - Providing telephone support lines - Cooperation between health organizations and non-governmental organizations which defend women - Financial and shelter support for women, especially refugees - Emergency accommodation for homeless women - Providing healthcare recommendations during the COVID-19 pandemic in the local languages - Providing individual sexual, physical, and psychological care services - Training psychologists to provide basic services to victims
Roy (2021) [6]	the UK	literature review	Physical, sexual, verbal, psychological, economic	- Quarantine and social distancing and the resulting stress - Economic disorder, unemployment, and poverty - No basic regulations - Insignificant social support - Not paying attention to women’s health needs - The development of societies and cultural and social prejudices - Lack of access to medical care and psychosocial support - The digital gap caused by virtual education	- Employment and income for women - The priority of governments to protect women - Providing primary care - Providing support services (telephone and internet lines, counseling, shelter and support centers for women under gender violence, social networks) - Tele-medicine - international humanitarian organizations Support - Use of previous pandemic experiences - Increasing awareness through social networks - Police cooperation through counseling
John (2020) [18]	USA	literature review	-	- Quarantine - No access to services required by women and girls - Economic problems of families - police non-intervention and imprisonment of the aggressors - Converting women’s shelters into shelters for the homeless - Gender inequality due to social norms	- Providing online and telephone consulting services - Holding trials online - Providing tele-medicine services - Providing guidance and training by women’s advocacy organizations nationally and internationally - Integrate the provision of gender services in critical situations - Women’s participation in decisions related to women’s affairs - Recording and collecting data based on gender and age in times of crisis
Jatmiko (2020) [19]	Indonesia	Qualitative	- Domestic violence - Online gender violence - Sexual harassment - Rape - Dating violence - Physical violence - Sexual-psychological violence - Women’s Suffering - Deprivation in the community or private life	- Quarantine and social distancing - Patriarchal culture - Illegal pornography trade in cyberspace	-
Donato (2020) [20]	Italy	Qualitative	Physical, mental, sexual, suicide	- Quarantine	- Preparing brochures and protocols to identify and deal with cases of gender-based violence - Centers for the protection of women under sexual violence - Psychological support - Government financial support
Afu (2020) [21]	Nigeria	Survey	Physical, sexual, psychological, Restricting women’s freedom	- alcohol consumption - Quarantine - Anti-woman social norms - Financial Problems	- Providing marriage counseling services - Providing counseling services for female adolescents in schools

### Psychometric evaluation

In this section, we answer the research questions and then recommend main strategies for combating gender-based violence against women in future pandemics.

### Dimensions of gender-based violence against women during the COVID-19 pandemic

The review of the literature showed that GBV against women is more in the form of verbal, emotional, psychological, physical, sexual, family, structural (government and community), economic, inheritance, online and dating, access to health, deprivation of liberty in community and personal life, femicide, and ultimately suicide.

Yenilmez stated that during the COVID-19 pandemic, the most important forms of GBV against women were physical, psychological, and sexual violence by their sexual partners, so that one in three women has faced this violence. Home quarantine is one of the factors that have contributed to this issue [4, 22]. Violence against women can be structural and direct. Structural violence is created through social and governmental laws and makes women economically dependent on men, while direct violence is related to family relationships and in the form of sexual violence and death. There are six types of GBV against women in Colombian society, including sexual, physical, psychological, inheritance, economic, and digital. This violence occurs at the level of family, workplace, university, community, organization, and in the form of genocide. In the private sector, this violence is emotional, physical, economic, and sexual, occurring in the scientific environment, workplace, community, and family. Violence against women is not just a matter of appearance; it is also in the form of placing them second to men. Violence against women in countries like Colombia and Mexico is not just related to family relations or marriage; it also includes criminal and drug-related violence. Thus, violence is divided into two categories: sexual partner violence and non-sexual partner violence [23].

Magezi stated that most GBV against women is in the form of physical, psychological, economic, and sexual violence perpetrated by sexual partners. Synonymous terms for violence by an emotional partner include “wife beating “, “beating”, and “domestic violence” [24]. The GBV is defined by Jatmiko as physical, sexual-psychological violence, suffering and social exclusion. Social networks created another type of gender-based violence as online gender-based violence. The justification for this online GBV is institutionalized patriarchy in cyberspace. This culture naturalizes GBV against women and ignores men’s mistakes in the form of online rape. Many cases of online gender-based violence include; virtual chats, rape videos, sex chat, taking photos of model women and broadcasting them in cyberspace or threatening to broadcast them if they do not have sex, and selling pornographic photos as an illegal trade have been reported by women [25].

### Factors affecting the incidence of gender-based violence during the COVID-19 pandemic

According to the literature, the most important factors affecting the incidence of GBV against women during the COVID-19 pandemic were quarantine and social distancing and the resulting stress; lack of access to social support for women and girls, women’s employment in the private and informal sectors; gender inequality and patriarchal social norms; failure to investigate cases of GBV by police and not prosecuting criminals, economic problems due to quarantine and unemployment of men, women’s unemployment and women’s economic dependence on men, women’s employment; alcohol and substance abuse by sexual partners and spouses, the digital gap in e-learning and access to social networks, the lack of transparent rules and registration system of real cases of GBV against women, age and level of education of women; no basic government regulations; the perception of violence against women as hallmark by the family and society, previous abusive relationships; dependence on children, threatening to kill women and children by men and not prosecuting online criminals. GBV against women increased with the beginning and continuation of quarantine and social distancing in COVID-19 pandemic [4, 22, 24, 26–30]. Violence against women is more common in public [3, 26], but during the quarantine, gender-based violence against women was more prevalent in the home and less in public. This has imposed a great psychological burden on women [24, 27–29]. No accurate reporting of violence against women causes does not receive much attention from governments. Women themselves may refuse to report it. Irregular distribution of this phenomenon in the whole population also causes the “classical elusive phenomenon”, which will affect the data collection. Violence against women is stigmatized by the family and society, and women refuse to report it [23]. The absence of clear internal rules and registration systems for GBV causes the lack of knowledge about the real cases of GBV against women and the confrontation of governments with it [3, 27].

The presence of men in the home does not mean their participation in household chores; men’s lack of cooperation in household chores, raising children and caring for the disabled and the elderly has imposed a great psychological burden on women and prevented them from earning money. Also, the laws and patriarchy have influenced the employment of women, especially during COVID-19 pandemic. So, women cannot play their political, social, and economic roles [22, 23]. Women horizontal inequality includes political inequality, inequality in access to education, and fertility rates that can lead to economic inequality and consequently violence against women. In terms of gender, this inequality places women in the minority group. Thus, the economic, social, and cultural implications of COVID-19 have made women the main victims of this pandemic [23].

Valencia et al. reported that declining political and economic role of women, unfavorable working conditions, women economic dependence on men, have led to sexual, physical, and psychological violence against women and even their death in the public and private sectors. So, government and social laws have encouraged this violence [23]. Lack of attention to menstrual health needs during quarantine in developing societies has also affected sexual health and reproduction in vulnerable women. The closure of schools during the pandemic and the online education has caused many problems for female students. Marginalization, mental health problems, digital gap, the stress of working at home, and uncertainty about the future affected female students during the pandemic. Also, quarantine financial pressures on families led to girls’ employment or early marriage [23].

Lack of services such as women’s rights network, health workers and teachers, in the pandemic lockdown has led to an increase in violence. Womens’ working in the informal sector reduces access to care and treatment facilities [4, 23]. Magezi stated that the weak economy, alcohol consumption at home, lack of access to socio-psychological support from religious and non-religious counselors, relationship with the abusive partner and staying with him for a long time during quarantine, lack of income and stress and economic dependence on spouse have led to an increase in GBV during the pandemic in Zimbabwe. In this country, most GBV is perpetrated by spouses. These women are eventually killed or commit suicide due to emotional depression, restrictions and beating. Concerns about children and fear of losing them due to divorce, lack of income, separation from family and friends, lack of access to social resources and religious centers, and lack of support from them, cultural and religious concepts instilled in women that they are inferior to men, threatening to kill their wives or children or themselves, as well as threatening women to leave home or exposing their husbands’ abuse, are factors associated with the increase of GBV against women during the COVID-19 pandemic [24].

Lund et al. reported that lack of access to shelters due to their conversion into medical centers or closure to prevent the spread of the disease is reason for the increase of the GBV [27]. In a study by Afu, quarantine and being long hours at home, economic problems, anti-woman norms in society, and alcohol consumption were presented as the main causes of GBV against women in Nigeria [31]. John et al. also reported that quarantine ,lack of access to services needed by women and girls and the use of existing services to prevent the transmission of COVID-19, economic problems of families, lack of police intervention and imprisonment of aggressors due to fears of outbreaks, turning the women’s shelter into a shelter for homeless people, the lack of accommodation for new people in the women’s shelter due to concerns about the spread of the disease and expressed anti-woman social norms as the factors that increase GBV against women [30]. Speed et al. stated that essential reasons for gender-based violence against women are the result of inequality of women’s power due to patriarchal social structures. Also, the stress, anxiety, and economic strain caused by a pandemic can contribute to this. Drug and alcohol use, inability to support family expenses, isolation of women, and overcrowding during the quarantine have increased GBV. The closure of asylums and non-accepting new people in accordance with government policies or at the request of the residents of these settlements has reduced the access of victims of sexual violence to these places during COVID-19 pandemic. This will have an adverse effect due to the increase in domestic violence cases and the decrease in the budget for refugees. As a result, the income of support organizations will decrease and their performance will be unfavorable in the future [3]. Lack of access to communication devices such as mobile phones and Internet are other aggravating factors [4].

### Research and practice recommendations

#### Social support

Marceline Naudi, chair of the Council of Europe’s Group of Experts declared that guidelines are among the most important ways to reduce GBV against women. Examples of these guidelines were developed by the European Convention on Prevention and Control in Turkey. Providing welfare and support services such as information centers and active helplines, including the numbers and addresses of local caregivers, and raising awareness among young women and girls are other ways to combat violence against women during pandemics. Italy, for example, used the number “1522” as a helpline for victims of sexual violence during the COVID-19 pundemic. Providing support lines in Australia, France, and the United Kingdom are another cases. Providing anti-violence policies is another approach used by France, Italy, and Spain. Providing shelter for victims, such as hotels, and providing care and accurate recording of violence against women have been other ways of combating GBV. In Italy, according to the law, criminals must leave the family, not the victims. Canada has also announced Sexual Victim Care Centers as part of its support package [4, 22]. Colombia has also set up special hotlines to record and track GBV against women. The calls are usually made to the public emergency. It is necessary to teach young women and girls in urban and rural areas how to use these lines. Women’s advocacy movements can also be effective in exposing violence against women and strengthening existing policies [23].

Establishing women’s advocacy centers in the form of legal, psychological, and health protections, establishing transparent government laws to protect women during pandemics, establishing local support systems, reviewing court rulings, and strengthening social support networks for families could be effective for women under GBV [22, 23]. The provision of these services should be based on maintaining the confidentiality of victims’ information and increasing their ability and life expectancy of their children. With a comprehensive approach, governmental and non-governmental organizations can work together to provide these services [32]. For example, UNICEF uses the AAAQ (Availability, Accessibility, Acceptability, and Quality) framework to measure victims’ access to social services through some questions. Supporting organizations should report the list of types of violence and lines of communication through websites, radio, television, and social networks [28].

Due to the impact of alcohol consumption on violence against women, South Africa has banned the sale of alcohol in clubs. Another strategy to support women was to provide support packages for women during COVID-19 pandemic, so-called “dignity kits”. People in the community should feel responsible for each other and receive the necessary training in this regard [4, 22]. Supporting organizations should increase the number of hours and days of service delivery and inform about this. These services include social support (food packages, emergency housing payments for families and providing toy packages for children), provision of educational packages for schools and training individuals and organizations that may be contacted by victims during COVID-19 pandemic [3, 32].

Community-based services can be effective in improving gender roles and determining the extent and nature of gender-based violence. The provision of refugees support services, such as accommodation in hotels and hospitals, has been provided by countries such as France, the United Kingdom, and Germany. Speed mentioned the establishment of a suitable accommodation for refugees and performing COVID-19 diagnostic tests, a 6 to 12-month support services after quarantine, and preparing a guide for appearing in court and police cooperation [3]. Humanitarian organizations can also provide services and collect the necessary data [32]. Churches can support women and even change men’s behavior by providing spiritual support [24]. Emergency housing for homeless women, providing individual sexual, physical, and psychological care services, training psychologists to provide basic services to victims, developing protocols and guidelines for combating GBV, women’s violence support centers in the form of psychological support, providing counseling services to couples on the adverse effects of GBV, educating and holding training seminars for girls in schools on marriage and the causes of sexual violence, and the importance of marital relations compared to other relations and trying to maintain it were stated as the most important strategies to deal with GBV [29, 31, 33].

### Healthcare

Providing online counseling services and creating a safe environment can be effective in promoting women’s mental and psychological health. Also, health workers and psychologists need to be trained and equipped [4, 22]. Caregivers must also have the skills, knowledge, and patience to help victims. Health care professionals need to be aware of the dangers and consequences of GBV and provide the necessary patient care, such as post-traumatic care. For older, disabled, poor women and minority, it is better to use tele-medicine [32]. Cooperation between health organizations and non-governmental organizations to support women in order to protect women who are under gender-based violence is also necessary [27]. Providing tele-medicine services is helpful to assist women and girls in preventing pregnancy or miscarriage, as well as providing online guidance and training for them [30].

### Government support

Amendments to government laws protecting the family must be considered. These laws include electronic control, the suspension of prison sentences, the creation of online crime registration and payment portals, the creation of virtual services to support sexually abused women, financial support for women, and women’s participation in charity [4, 22]. Governments also need to prioritize their work and provide services to victims [28]. Governments’ planning to support women and create a safe work environment for them is necessary. Government actions include the provision of primary care, efficient and adequate health care infrastructure and manpower, adequate resources, and support services (telephone and Internet lines, counseling, and shelters for women subjected to gender-based violence). Governments need to learn from previous pandemic experiences and find ways to reduce the harm to women in future pandemics. Police cooperation is another solution by providing advice and creating a safe space in the house without the access of intruders [32]. Cooperation and participation of governmental and non-governmental organizations at the national and international levels such are other solutions used. Providing homework assignments during the pandemic, food and social security programs, especially for bisexuals who are more prone to gender-based violence are among solutions used [24]. In the United Kingdom, the government supported GBV organizations during COVID-19 pandemic by allocating financial packages. Evidence shows that providing funding and a financial recording system for small victim advocacy organizations can be helpful. Government financial support for women can take the form of employment and participation in charity activities, such as making face mask [4, 22]. In Bangladesh, evidence has shown that households who offered interest-free loans to expedite men immigration have reduced spouses’ physical and sexual violence by 3.5% during six months. Also, women’s employment and income can reduce financial dependence and violence against them. Therefore, governments need to plan to support women and create a safe work environment for them. The use of evidence-based programs and policies to support women entrepreneurship and advocacy organizations is another strategy [32].

### Information and communication technology

Using the code in France, Italy, and Spain for reporting suspicious cases is a protective measure. The covert mobile apps to show signals that abusive people are close has been used in the United Kingdom and Italy. Working with these mobile-based applications should be easy and the incident can be reported to the police with just one click [4, 22]. Another option was to set up pop-up booths by organizations supporting women under GBV in supermarkets and pharmacies. In Colombia, creating a safe space for victims in supermarkets and pharmacies in the form of telephone counseling rooms was on the agenda. These hotlines have been used for forced marriage counseling, sexual violence, men’s counseling, and domestic violence counseling in the United Kingdom. Maintaining confidentiality and privacy in the consulting lines particularly in the web environment is necessary [3]. Supporting organizations are advised to provide a list of violence types and ways of communication through websites, radio, television, and social media [28, 33]. The Home Secretary’s “You Are Not Alone” campaign was another initiative in the UK. The campaign was designed to inform at-risk individuals so that they can access to support services and the police. Other initiatives included providing online victim support and Fujitsu security support to provide IT infrastructure for smaller charities. Remote court services can also be used to hear statements made by victims of gender-based violence. The service was used in the UK via Skype and the cloud video platform. Complaints and follow-up guidelines should be posted on the websites of organizations supporting women victims of gender-based violence, legal centers, and social media. Security and definition of access on these platforms is important. The police can also receive the statements of victims and witnesses by phone and receive confirmation of the statements by electronic or non-electronic signature via email or mail. Also, virtual interviews or virtual and face-to-face interviews can be used depending on the importance of the issue. Many women are also uncomfortable holding these sessions at home due to the presence of children; in this case, it is necessary that family courts to be held in the short session. Information on courts and available legal centers during the pandemic should to be provided. Support counseling in the form of online services is of particular importance [3, 27, 30, 32]. An example of such services is CEPAM-Guayaquil Telephone Consulting in Ecuador. In Italy, the National Network of Domestic Violence Shelters has provided support services via Skype and telephone [30].

**Fig. 2:**
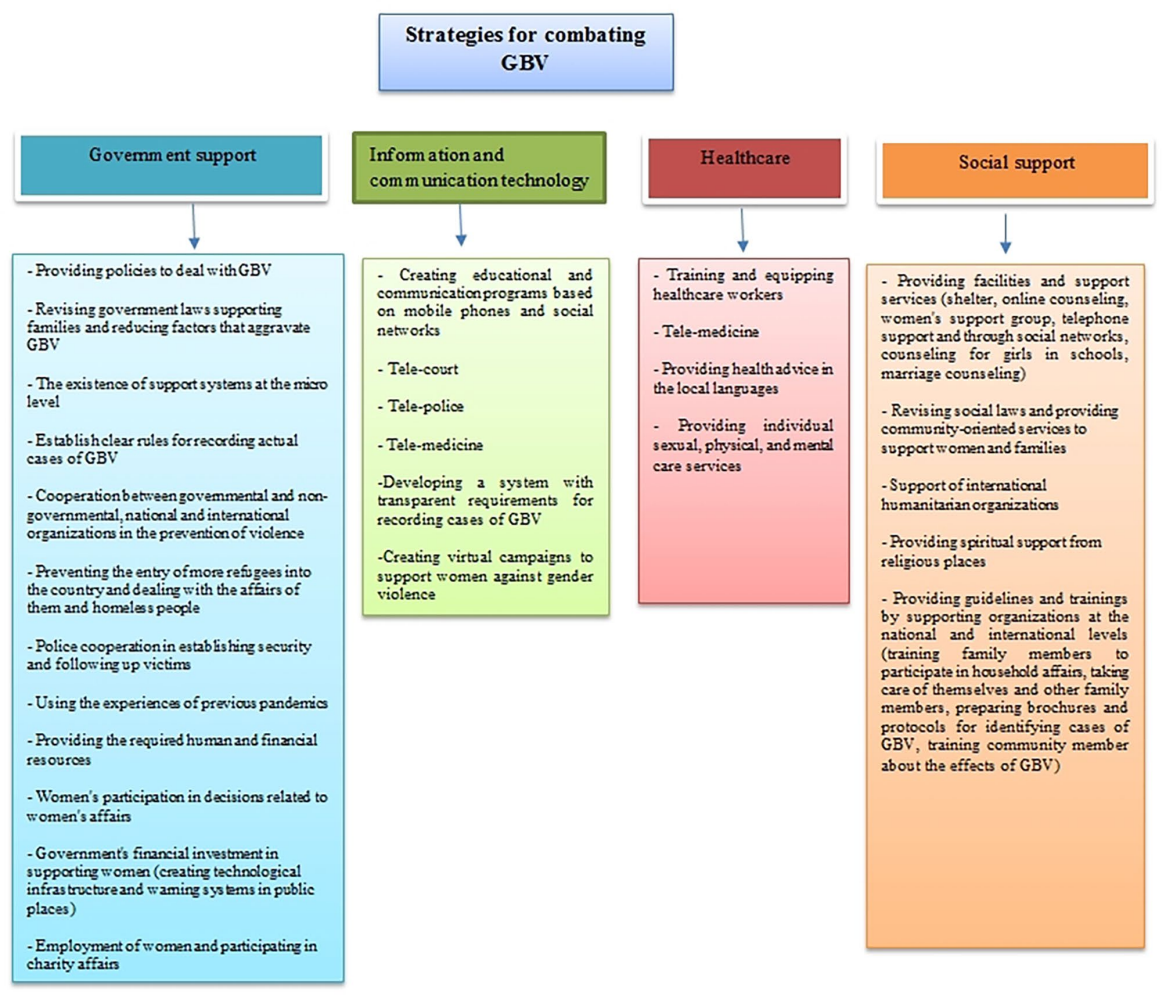
Recommended strategies for managing GBV against women in future pandemics is as follows

Launching campaigns to combat GBV against women, such as the “Red Mask” campaign, was another strategy. The campaign was launched first in Spain, then in France, Chile, and Argentina under the code “Mask 19”. Argentina used WhatsApp, mobile, and emails to connect more with victims. Public and private campaigns prevented more victims from being at risk by using silent methods such as codes. Another campaign was to raise awareness among rapists about the consequences of GBV. But there was no national system for accessing resources. Access to information on actions taken by governmental and non-governmental organizations to combat gender-based violence can contribute to evidence-based policies [28]. Creating an accessible service database for victims of gender-based violence is essential. An example of this database in the UK is the “Companies House or the Charity Commission”. This database provides easy access to services for victims and payment to organizations in a convenient way. Holding webinars and online training programs can affect the staff cooperation of sponsoring organizations. Community-based services can also be effective in improving healthy gender roles and determining the extent and nature of GBV. Speed suggested government support for educating charities to attend online and provide training programs for victims [3]. In order to get acquainted with the needs and barriers to providing services to them, women also should be involved in service decisions. Collected data during the crisis should be segregated by age and gender [30]. Based on this scoping review, we recommended strategies for managing GBV against women in future pandemics is as follows (Fig. 2).

## Discussion

In this scoping review, we provided a comprehensive synthesis of the published literature on GBV against women in the COVID-19 era. Results of this work showed, the COVID-19 as an emerging disease spread rapidly, and then many problems arose for the people worldwide, one of these challenges was the increase of violence against women. Dealing with GBV against women required identifying its dimensions, effective factors on it, and strategies to reduce it, which was done in the present study.

We found that the most common GBV against women were physical, sexual, psychological, emotional, economic, digital and virtual, substance use, structural (society and law), verbal, deprivation in personal or social life, femicide and suicide. Stark study showed that the common forms of GBV were sexual partner violence, physical violence, sexual violence, and rape that it mostly happens outside the house [34]. In a study by Mittal, the most common forms of GBV were physical, sexual, emotional, domestic and female genital mutilation which was similar to this study [16]. Results of other study showed that most common types of violence against women are psychological/verbal, physical, and sexual, respectively. There was a significant relationship between couples’ age gap, forced marriage, husband addiction, income, and history of violence experienced by the husband with domestic violence against women [35]. Also, study in Uganda reveals several factors associated with increased risk and vulnerability to GBV during COVID19. Socio-economic status particularly linked to low education achievement (primary education) and the need for assistance to access health care was associated with higher likelihood to experience increased risk and vulnerability to GBV [2].

These studies showed that the incidence of violence against women had increased due to quarantine conditions and social distancing during the COVID-19 pandemic.

The most GBV before COVID-19 occurred outdoors. In other words, the type of GBV against women during the quarantine is different from before, this can be due to the presence of men at home for more times because of job loss and mental and emotional distress caused by economic problems. Also, the difference between types of GBV among countries can be caused by cultural differences and their level of development. This requires the development of livelihood packages and financial assistance managed by the policy makers of a country.

A review of the existing literature showed that the most common factors influencing the incidence of GBV against women in the COVID-19 era were: lack of access to social support for women and girls, women’s employment in the private and informal sectors, gender inequality and patriarchal social norms, failure of the police to deal with cases of gender-based violence and the prosecution of perpetrators, economic problems due to quarantine and unemployment of men, women’s unemployment and women’s economic dependence on men, alcohol and substance abuse by sexual partners and spouses, the digital gap in e-learning and access to social networks, lack of clear laws and a system for recording real cases of gender-based violence against women, age and level of education of women, lack of basic government regulations, the perception of violence against women as hallmark by the family and society, previous abusive relationships, dependence on children and failure to prosecute online perpetrators of gender-based violence. However, quarantine has been effective in reducing disease transmission, but because of job losses, economic and psychological problems, loneliness and insecurity violent behaviors such as gender-based violence against women have increased. In Mittal study, they pointed out the lack of accurate reporting of cases of GBV during the pandemics, which shows that countries do not pay enough attention to this issue in critical situations [16]. In addition, results of other studies revealed an association between female sex and more risk for burnout [36]. Based on literature, females have a tendency to be more susceptible to experiencing the signs of stress particularly, nurses [37].

Therefore, the establishment of comprehensive national systems for recording and addressing cases of GBV should be on the agenda of governments.

Besides, the laws and traditions that govern society are other causes of GBV against women. Patriarchal societies place women second to men, and this provides the basis for all kinds of GBV against them [38]. In order to reduce GBV against women, it is suggested that in traditional societies where women have less freedom, arrangements should be made to educate them from school age so that they become familiar with their basic rights. It is necessary that supporting legal institutions follow up and deal with any violence in countries. These trainings should not be limited only to women, but it needs to take action in the whole society regarding any kind of violence against women, and every person should consider himself responsible in this regard. These necessities must be prepared before any crisis, especially pandemics. The issue of government financial support should also be paid to the attention of countries in order to create a sense of financial security in difficult situations. The lack of financial security can lead to all kinds of violence in society, the majority of which will be directed at women.

Arthur and Clark stated that one of the reasons for the increase in GBV against women is their economic dependence on men, which is exacerbated by quarantine due to women’s employment in the private and informal sectors [39]. Also, women have fewer remote working conditions than men, which make it difficult for them to adapt [14]. This not only increases the risk of GBV but also makes it impossible to leave spouses and sexual partners. Accordingly, it is necessary for governments to pay attention to the financial needs of women in a pandemic situation and put women distance working on their agenda to reduce their financial dependence on their spouse. The increase in refugees due to the economic problems caused by the pandemic will cause them to be unable to meet their daily needs and as a result the GBV against them will increase. This requires countries’ policies and financial planning to control and reduce the influx of refugees to manage their access to health care services, psychological counseling, courts, police and housing.

On the other hand, outbreaks of pandemics such as influenza, swine flu, and SARS have caused psychological problems in the form of substance and alcohol use, anxiety, and depression that have persisted since the end of the pandemic [40, 41]. Results of systematic review showed that the prevalence of postpartum depression in women was relatively high during COVID-19 [42].

These psychological problems caused by pandemics lead to a variety of violence such as gender-based violence [43]. Therefore, it is necessary to provide psychological health care services and training for resilience in such situations for women and young girls. Due to pandemic conditions it is better to provide these services in the form of distance counseling and education. However, gender-based violence against women is not limited to pandemic conditions; it also occurs in natural disasters such as floods, earthquakes, and hurricanes [44, 45]. According to the Yasmin study, cases of sexual violence in the form of rape and sexual abuse increased significantly during the Ebola outbreak in South Africa [46]. Given the distinction between different crises caused by natural disasters, war, or the prevalence of diseases and their specific characteristics, it needs to conduct studies to provide a model and solution to combat violence against women.

This review showed that during the COVID-19 pandemic, various measures have been taken to combat and reduce GBV against women by countries, which can be a guide for similar events in future pandemics. The main strategies were divided into five categories: government support in the form of policies and planning, social and humanitarian institutions support, government economic support, health organizations support and using cyberspace to provide health care, remote courts, remote police, online counseling and training of under GBV women. According to Campell, the most important step in combating gender-based violence is to raise people awareness about the importance of gender-based violence [44]. Other effective measures are the establishment of communication channels through telephone and the Internet to report cases of gender-based violence, counseling, training and follow-up [47]. The current study revealed the emphasis of the studies on this issue. However, educating individuals should not be limited to victims; rather, all members of society should be responsible, report cases of gender-based violence and help victims voluntarily [48]. Health care providers should also be trained in identifying and addressing symptoms of violence and taking effective and timely action for victims [49]. Social media can also help to educate victims by publishing guidelines, advertisements and raising awareness [50]. This review showed that legal and accountable centers such as the police and medical and social support centers can publish details of access to services through their websites or social networks and help victims. Another measure is the establishment of emergency hotline to support victims. Various studies have pointed to the training and use of staff who are specialized in psychology, psychiatry, social, and legal services, as well as emergency alert systems in grocery stores and pharmacies, cloud and online platforms, online text chat, online courts, shelters, policy-making and government funding [16, 51]

Overall, one of the strengths of this study was to provide a comprehensive perspective on the dimensions of GBV against women, factors affecting it, and ways to deal with it during the COVID-19 era, which had not been done before in the form of a scoping review.

### Limitations

However, this study also had some limitations that one of them was the lack of access to the full-text of numerous studies, which was attempted to be accessed through correspondence with their authors on social networks. Another limitation of this study was not including studies such as proceeding papers, perspectives, commentaries, articles and grey literature in other languages. Also, the lack of quality assessment of the studies was another limitation of this research, which was not done because of different methodology of the included studies. Therefore, due to the lack of serious attention and almost ignoring the issue of gender-based violence against women in critical situations such as pandemics, it is suggested that researchers in different countries investigated effect of the recommended strategies to combat gender-based violence that can be used in future pandemics and crises.

## Conclusion

The results showed that GBV against women accrues more in the form of verbal, emotional, psychological, physical, sexual, family, structural, economic, inheritance, online and dating, access to health, deprivation of liberty in community and personal life, femicide, and suicide. Various factors affected the occurrence of GBV against women during the COVID-19 era such as quarantine and social distancing, lack of access to social support, women’s employment in the private and informal sectors; gender inequality, economic problems, alcohol and substance abuse, the digital gap, no basic government regulations, etc. It is suggested that countries provide sufficient ICT infrastructure, comprehensive policies and planning, economic support, social support by collaboration between national and international organizations, and healthcare supporting to manage incidence of GBV against women in future pandemics. The consequences of GBV for its victims are long-lasting and rampant for responses that are often inadequate. Hence, it is important to maintain urgency in cases of GBV even in critical situations. Based on the results of the reviews, the need for a comprehensive response model to address the issue of gender-based violence during current and possible future pandemics is essential. The opinions of health professionals, formal and informal media, and community efforts must be combined to effectively address the issue of gender-based violence. Additionally, continued and serious efforts are needed to end the stigma associated with gender-based violence.

## Electronic supplementary material

Below is the link to the electronic supplementary material.

Additional File: Search strategies

## Data Availability

The data that support the findings of this study are available from the corresponding author but restrictions apply to the availability of these data, which were used under license for the current study, and so are not publicly available. Data are however available from the authors upon reasonable request and with permission of the corresponding author.
