# Supplementary material for: Gender-based violence against women during the COVID-19 pandemic: recommendations for future
Source: BMC Womens Health. 2023 May 3;23:219. doi: 10.1186/s12905-023-02372-6 (PMC10155645; doi:10.1186/s12905-023-02372-6)
Supplement: Supplementary file 1 — Additional File: Search strategies [file 12905_2023_2372_MOESM1_ESM.docx]

Appendix 1. Search strategies

| Databases | Search strategy | Results |
| --- | --- | --- |
| PubMed | (Girl[tiab] OR Woman[tiab] OR women[tiab] OR “Women's Groups”[tiab] OR “Women Groups”[tiab] OR “Women's Group”[tiab] OR female[tiab]) AND (“Sex Offenses”[tw] OR (Offense[tiab] AND Sex[tiab]) OR (Offenses[tiab] AND Sex[tiab]) OR “Sex Offense”[tiab] OR “Sexual Violence”[tw] OR “Sexual Violences”[tiab] OR (Violence[tiab] AND Sexual[tiab]) OR (Violences[tiab] AND Sexual[tiab]) OR “Sexual Abuse”[tw] OR (Abuse[tiab] AND Sexual[tiab]) OR (Abuses[tiab] AND Sexual[tiab]) OR “Sexual Abuses”[tiab] OR “sexual aggression”[tw] OR “domestic violence”[tw] OR (Violence[tiab] AND Domestic[tiab]) OR “Family Violence”[tw] OR (Violence[tiab] AND Family[tiab]) “gender based violence”[tiab] OR violence[tiab] OR abuse[tiab] OR offense[tiab] OR aggression[tiab] OR domestic[tiab] OR sex[tiab] OR family[tiab]) AND (“COVID 19”[tiab] OR “COVID-19 Virus Disease”[tiab] OR “COVID 19 Virus Disease”[tiab] OR “COVID-19 Virus Diseases”[tiab] OR (Disease[tiab] AND “COVID-19 Virus”[tiab]) OR (“Virus Disease”[tiab] AND “COVID-19”[tiab]) OR “COVID-19 Virus Infection”[tiab] OR “COVID 19 Virus Infection”[tiab] OR “COVID-19 Virus Infections”[tiab] OR (Infection[tiab] AND “COVID-19 Virus”[tiab]) OR (“Virus Infection”[tiab] AND “COVID-19”[tiab]) OR “2019-nCoV Infection”[tiab] OR “2019 nCoV Infection”[tiab] OR “2019-nCoV Infections”[tiab] OR (Infection[tiab] AND “2019-nCoV”[tiab]) OR “Coronavirus Disease-19”[tiab] OR “Coronavirus Disease 19”[tiab] OR “2019 Novel Coronavirus Disease”[tiab] OR “2019 Novel Coronavirus Infection”[tiab] OR “2019-nCoV Disease”[tiab] OR “2019 nCoV Disease”[tiab] OR “2019-nCoV Diseases”[tiab] OR (Disease[tiab] AND “2019-nCoV”[tiab]) OR “COVID19”[tiab] OR “Coronavirus Disease 2019”[tiab] OR (“Disease 2019”[tiab] AND Coronavirus[tiab]) OR “SARS Coronavirus 2 Infection”[tiab] OR “SARS-CoV-2 Infection”[tiab] OR (Infection[tiab] AND “SARS-CoV-2”[tiab]) OR “SARS CoV 2 Infection”[tiab] OR “SARS-CoV-2 Infections”[tiab] OR “COVID-19 Pandemic”[tiab] OR “COVID 19 Pandemic”[tiab] OR “COVID-19 Pandemics”[tiab] OR (Pandemic[tiab] AND “COVID-19”[tiab]) OR “Coronavirus Disease 2019 Virus”[tiab] OR “2019 Novel Coronavirus”[tiab] OR “2019 Novel Coronaviruses”[tiab] OR (Coronavirus[tiab] AND “2019 Novel”[tiab]) OR (“Novel Coronavirus”[tiab] AND 2019[tiab]) OR “Wuhan Seafood Market Pneumonia Virus”[tiab] OR “SARS-CoV-2 Virus”[tiab] OR “SARS CoV 2 Virus”[tiab] OR “SARS-CoV-2 Viruses”[tiab] OR (Virus[tiab] AND “SARS-CoV-2”[tiab]) OR “2019-nCoV”[tiab] OR “COVID-19 Virus”[tiab] OR “COVID 19 Virus”[tiab] OR “COVID-19 Viruses”[tiab] OR (Virus[tiab] AND “COVID-19”[tiab]) OR “Wuhan Coronavirus”[tiab] OR (Coronavirus[tiab] AND Wuhan[tiab]) OR “SARS Coronavirus 2”[tiab] OR (“Coronavirus 2”[tiab] AND SARS[tiab]) OR “Severe Acute Respiratory Syndrome Coronavirus 2”[tiab]) | 1174 |
| Embase | (Girl:ti,ab OR Woman:ti,ab OR women:ti,ab OR “Women Groups”:ti,ab OR “Women Group”:ti,ab OR female:ti,ab) AND (“Sex Offenses”/exp OR (Offense:ti,ab AND Sex:ti,ab) OR (Offenses:ti,ab AND Sex:ti,ab) OR “Sex Offense”:ti,ab OR “Sexual Violence”/exp OR “Sexual Violences”:ti,ab OR (Violence:ti,ab AND Sexual:ti,ab) OR (Violences:ti,ab AND Sexual:ti,ab) OR “Sexual Abuse”/exp OR (Abuse:ti,ab AND Sexual:ti,ab) OR (Abuses:ti,ab AND Sexual:ti,ab) OR “Sexual Abuses”:ti,ab OR “sexual aggression”/exp OR “domestic violence”/exp OR (Violence:ti,ab AND Domestic:ti,ab) OR “Family Violence”/exp OR (Violence:ti,ab AND Family:ti,ab) OR”gender based violence”:ti,ab OR violence:ti,ab OR abuse:ti,ab OR offense:ti,ab OR aggression:ti,ab OR domestic:ti,ab OR sex:ti,ab OR family:ti,ab) AND (“COVID 19”:ti,ab OR “COVID-19 Virus Disease”:ti,ab OR “COVID 19 Virus Disease”:ti,ab OR “COVID-19 Virus Diseases”:ti,ab OR (Disease:ti,ab AND “COVID-19 Virus”:ti,ab) OR (“Virus Disease”:ti,ab AND “COVID-19”:ti,ab) OR “COVID-19 Virus Infection”:ti,ab OR “COVID 19 Virus Infection”:ti,ab OR “COVID-19 Virus Infections”:ti,ab OR (Infection:ti,ab AND “COVID-19 Virus”:ti,ab) OR (“Virus Infection”:ti,ab AND “COVID-19”:ti,ab) OR “2019-nCoV Infection”:ti,ab OR “2019 nCoV Infection”:ti,ab OR “2019-nCoV Infections”:ti,ab OR (Infection:ti,ab AND “2019-nCoV”:ti,ab) OR “Coronavirus Disease-19”:ti,ab OR “Coronavirus Disease 19”:ti,ab OR “2019 Novel Coronavirus Disease”:ti,ab OR “2019 Novel Coronavirus Infection”:ti,ab OR “2019-nCoV Disease”:ti,ab OR “2019 nCoV Disease”:ti,ab OR “2019-nCoV Diseases”:ti,ab OR (Disease:ti,ab AND “2019-nCoV”:ti,ab) OR “COVID19”:ti,ab OR “Coronavirus Disease 2019”:ti,ab OR (“Disease 2019”:ti,ab AND Coronavirus:ti,ab) OR “SARS Coronavirus 2 Infection”:ti,ab OR “SARS-CoV-2 Infection”:ti,ab OR (Infection:ti,ab AND “SARS-CoV-2”:ti,ab) OR “SARS CoV 2 Infection”:ti,ab OR “SARS-CoV-2 Infections”:ti,ab OR “COVID-19 Pandemic”:ti,ab OR “COVID 19 Pandemic”:ti,ab OR “COVID-19 Pandemics”:ti,ab OR (Pandemic:ti,ab AND “COVID-19”:ti,ab) OR “Coronavirus Disease 2019 Virus”:ti,ab OR “2019 Novel Coronavirus”:ti,ab OR “2019 Novel Coronaviruses”:ti,ab OR (Coronavirus:ti,ab AND “2019 Novel”:ti,ab) OR (“Novel Coronavirus”:ti,ab AND 2019:ti,ab) OR “Wuhan Seafood Market Pneumonia Virus”:ti,ab OR “SARS-CoV-2 Virus”:ti,ab OR “SARS CoV 2 Virus”:ti,ab OR “SARS-CoV-2 Viruses”:ti,ab OR (Virus:ti,ab AND “SARS-CoV-2”:ti,ab) OR “2019-nCoV”:ti,ab OR “COVID-19 Virus”:ti,ab OR “COVID 19 Virus”:ti,ab OR “COVID-19 Viruses”:ti,ab OR (Virus:ti,ab AND “COVID-19”:ti,ab) OR “Wuhan Coronavirus”:ti,ab OR (Coronavirus:ti,ab AND Wuhan:ti,ab) OR “SARS Coronavirus 2”:ti,ab OR (“Coronavirus 2”:ti,ab AND SARS:ti,ab) OR “Severe Acute Respiratory Syndrome Coronavirus 2”:ti,ab)=1187 | 1187 |
| Scopus | (TITLE-ABS(Girl) OR TITLE-ABS(Woman) OR TITLE-ABS(women) OR TITLE-ABS(“Women Groups”) OR TITLE-ABS(“Women Group”) OR TITLE-ABS(female)) AND (TITLE-ABS(“Sex Offenses”) OR (TITLE-ABS(Offense) AND TITLE-ABS(Sex)) OR (TITLE-ABS(Offenses) AND TITLE-ABS(Sex)) OR TITLE-ABS(“Sex Offense”) OR TITLE-ABS(“Sexual Violence”) OR TITLE-ABS(“Sexual Violences”) OR (TITLE-ABS(Violence) AND TITLE-ABS(Sexual)) OR (TITLE-ABS(Violences) AND TITLE-ABS(Sexual)) OR TITLE-ABS(“Sexual Abuse”) OR (TITLE-ABS(Abuse) AND TITLE-ABS(Sexual)) OR (TITLE-ABS(Abuses) AND TITLE-ABS(Sexual)) OR TITLE-ABS(“Sexual Abuses”) OR TITLE-ABS(“sexual aggression”) OR TITLE-ABS(“domestic violence”) OR (TITLE-ABS(Violence) AND TITLE-ABS(Domestic)) OR TITLE-ABS-KEY(“gender based violence”) OR TITLE-ABS(“Family Violence”) OR TITLE-ABS(violence) OR TITLE-ABS(abuse) OR TITLE-ABS(offense) OR TITLE-ABS(aggression) OR TITLE-ABS(domestic) OR TITLE-ABS(sex) OR TITLE-ABS(family)) AND (TITLE-ABS(“COVID 19”) OR TITLE-ABS(“COVID-19 Virus Disease”) OR TITLE-ABS(“COVID 19 Virus Disease”) OR TITLE-ABS(“COVID-19 Virus Diseases”) OR (TITLE-ABS(Disease) AND TITLE-ABS(“COVID-19 Virus”)) OR (TITLE-ABS(“Virus Disease”) AND TITLE-ABS(“COVID-19”)) OR TITLE-ABS(“COVID-19 Virus Infection”) OR TITLE-ABS(“COVID 19 Virus Infection”) OR TITLE-ABS(“COVID-19 Virus Infections”) OR (TITLE-ABS(Infection) AND TITLE-ABS(“COVID-19 Virus”)) OR (TITLE-ABS(“Virus Infection”) AND TITLE-ABS(“COVID-19”)) OR TITLE-ABS(“2019-nCoV Infection”) OR TITLE-ABS(“2019 nCoV Infection”) OR TITLE-ABS(“2019-nCoV Infections”) OR (TITLE-ABS(Infection) AND TITLE-ABS(“2019-nCoV”)) OR TITLE-ABS(“Coronavirus Disease-19”) OR TITLE-ABS(“Coronavirus Disease 19”) OR TITLE-ABS(“2019 Novel Coronavirus Disease”) OR TITLE-ABS(“2019 Novel Coronavirus Infection”) OR TITLE-ABS(“2019-nCoV Disease”) OR TITLE-ABS(“2019 nCoV Disease”) OR TITLE-ABS(“2019-nCoV Diseases”) OR (TITLE-ABS(Disease) AND TITLE-ABS(“2019-nCoV”)) OR TITLE-ABS(“COVID19”) OR TITLE-ABS(“Coronavirus Disease 2019”) OR (TITLE-ABS(“Disease 2019”) AND TITLE-ABS(Coronavirus)) OR TITLE-ABS(“SARS Coronavirus 2 Infection”) OR TITLE-ABS(“SARS-CoV-2 Infection”) OR (TITLE-ABS(Infection) AND TITLE-ABS(“SARS-CoV-2”)) OR TITLE-ABS(“SARS CoV 2 Infection”) OR TITLE-ABS(“SARS-CoV-2 Infections”) OR TITLE-ABS(“COVID-19 Pandemic”) OR TITLE-ABS(“COVID 19 Pandemic”) OR TITLE-ABS(“COVID-19 Pandemics”) OR (TITLE-ABS(Pandemic) AND TITLE-ABS(“COVID-19”)) OR TITLE-ABS(“Coronavirus Disease 2019 Virus”) OR TITLE-ABS(“2019 Novel Coronavirus”) OR TITLE-ABS(“2019 Novel Coronaviruses”) OR (TITLE-ABS(Coronavirus) AND TITLE-ABS(“2019 Novel”)) OR (TITLE-ABS(“Novel Coronavirus”) AND TITLE-ABS(2019)) OR TITLE-ABS(“Wuhan Seafood Market Pneumonia Virus”) OR TITLE-ABS(“SARS-CoV-2 Virus”) OR TITLE-ABS(“SARS CoV 2 Virus”) OR TITLE-ABS(“SARS-CoV-2 Viruses”) OR (TITLE-ABS(Virus) AND TITLE-ABS(“SARS-CoV-2”)) OR TITLE-ABS-KEY(“2019-nCoV”) OR TITLE-ABS(“COVID-19 Virus”) OR TITLE-ABS(“COVID 19 Virus”) OR TITLE-ABS(“COVID-19 Viruses”) OR (TITLE-ABS(Virus) AND TITLE-ABS(“COVID-19”)) OR TITLE-ABS(“Wuhan Coronavirus”) OR (TITLE-ABS(Coronavirus) AND TITLE-ABS(Wuhan)) OR TITLE-ABS(“SARS Coronavirus 2”) OR (TITLE-ABS(“Coronavirus 2”) AND TITLE-ABS(SARS)) OR TITLE-ABS(“Severe Acute Respiratory Syndrome Coronavirus 2”)) | 1495 |
| Web of Science | (TS=(Girl) OR TS=(Woman) OR TS=(women) OR TS=(“Women Groups”) OR TS=(“Women Group”) OR TS=(female)) AND (TS=(“Sex Offenses”) OR (TS=(Offense) AND TS=(Sex)) OR (TS=(Offenses) AND TS=(Sex)) OR TS=(“Sex Offense”) OR TS=(“Sexual Violence”) OR TS=(“Sexual Violences”) OR (TS=(Violence) AND TS=(Sexual)) OR (TS=(Violences) AND TS=(Sexual)) OR TS=(“Sexual Abuse”) OR (TS=(Abuse) AND TS=(Sexual)) OR (TS=(Abuses) AND TS=(Sexual)) OR TS=(“Sexual Abuses”) OR TS=(“sexual aggression”) OR TS=(“domestic violence”) OR (TS=(Violence) AND TS=(Domestic)) OR TS=(“gender based violence”) OR TS=(“Family Violence”) OR TS=(violence) OR TS=(abuse) OR TS=(offense) OR TS=(aggression) OR TS=(domestic) OR TS=(sex) OR TS=(family)) AND (TS=(“COVID 19”) OR TS=(“COVID-19 Virus Disease”) OR TS=(“COVID 19 Virus Disease”) OR TS=(“COVID-19 Virus Diseases”) OR (TS=(Disease) AND TS=(“COVID-19 Virus”)) OR (TS=(“Virus Disease”) AND TS=(“COVID-19”)) OR TS=(“COVID-19 Virus Infection”) OR TS=(“COVID 19 Virus Infection”) OR TS=(“COVID-19 Virus Infections”) OR (TS=(Infection) AND TS=(“COVID-19 Virus”)) OR (TS=(“Virus Infection”) AND TS=(“COVID-19”)) OR TS=(“2019-nCoV Infection”) OR TS=(“2019 nCoV Infection”) OR TS=(“2019-nCoV Infections”) OR (TS=(Infection) AND TS=(“2019-nCoV”)) OR TS=(“Coronavirus Disease-19”) OR TS=(“Coronavirus Disease 19”) OR TS=(“2019 Novel Coronavirus Disease”) OR TS=(“2019 Novel Coronavirus Infection”) OR TS=(“2019-nCoV Disease”) OR TS=(“2019 nCoV Disease”) OR TS=(“2019-nCoV Diseases”) OR (TS=(Disease) AND TS=(“2019-nCoV”)) OR TS=(“COVID19”) OR TS=(“Coronavirus Disease 2019”) OR (TS=(“Disease 2019”) AND TS=(Coronavirus)) OR TS=(“SARS Coronavirus 2 Infection”) OR TS=(“SARS-CoV-2 Infection”) OR (TS=(Infection) AND TS=(“SARS-CoV-2”)) OR TS=(“SARS CoV 2 Infection”) OR TS=(“SARS-CoV-2 Infections”) OR TS=(“COVID-19 Pandemic”) OR TS=(“COVID 19 Pandemic”) OR TS=(“COVID-19 Pandemics”) OR (TS=(Pandemic) AND TS=(“COVID-19”)) OR TS=(“Coronavirus Disease 2019 Virus”) OR TS=(“2019 Novel Coronavirus”) OR TS=(“2019 Novel Coronaviruses”) OR (TS=(Coronavirus) AND TS=(“2019 Novel”)) OR (TS=(“Novel Coronavirus”) AND TS=(2019)) OR TS=(“Wuhan Seafood Market Pneumonia Virus”) OR TS=(“SARS-CoV-2 Virus”) OR TS=(“SARS CoV 2 Virus”) OR TI=(“SARS-CoV-2 Viruses”) OR (TI=(Virus) AND TI=(“SARS-CoV-2”)) OR TI=(“2019-nCoV”) OR TS=(“COVID-19 Virus”) OR TS=(“COVID 19 Virus”) OR TS=(“COVID-19 Viruses”) OR (TS=(Virus) AND TS=(“COVID-19”)) OR TS=(“Wuhan Coronavirus”) OR (TS=(Coronavirus) AND TS=(Wuhan)) OR TS=(“SARS Coronavirus 2”) OR (TS=(“Coronavirus 2”) AND TS=(SARS)) OR TS=(“Severe Acute Respiratory Syndrome Coronavirus 2”)) | 1345 |
| Proquest | TI,AB,SU(Girl OR Woman OR women OR “Women's Groups” OR “Women Groups” OR “Women's Group” OR female) AND TI,AB,SU(“Sex Offenses” OR (Offense AND Sex) OR (Offenses AND Sex) OR “Sex Offense” OR “Sexual Violence” OR “Sexual Violences” OR (Violence AND Sexual) OR (Violences AND Sexual) OR “Sexual Abuse” OR (Abuse AND Sexual) OR (Abuses AND Sexual) OR “Sexual Abuses” OR “sexual aggression” OR “domestic violence” OR (Violence AND Domestic) OR “Family Violence” OR (Violence AND Family) OR “gender based violence” OR violence OR abuse OR offense OR aggression OR domestic OR sex OR family) AND TI,AB,SU(“COVID 19” OR “COVID-19 Virus Disease” OR “COVID 19 Virus Disease” OR “COVID-19 Virus Diseases” OR (Disease AND “COVID-19 Virus”) OR (“Virus Disease” AND “COVID-19”) OR “COVID-19 Virus Infection” OR “COVID 19 Virus Infection” OR “COVID-19 Virus Infections” OR (Infection AND “COVID-19 Virus”) OR (“Virus Infection” AND “COVID-19”) OR “2019-nCoV Infection” OR “2019 nCoV Infection” OR “2019-nCoV Infections” OR (Infection AND “2019-nCoV”) OR “Coronavirus Disease-19” OR “Coronavirus Disease 19” OR “2019 Novel Coronavirus Disease” OR “2019 Novel Coronavirus Infection” OR “2019-nCoV Disease” OR “2019 nCoV Disease” OR “2019-nCoV Diseases” OR (Disease AND “2019-nCoV”) OR “COVID19” OR “Coronavirus Disease 2019” OR (“Disease 2019” AND Coronavirus) OR “SARS Coronavirus 2 Infection” OR “SARS-CoV-2 Infection” OR (Infection AND “SARS-CoV-2”) OR “SARS CoV 2 Infection” OR “SARS-CoV-2 Infections” OR “COVID-19 Pandemic” OR “COVID 19 Pandemic” OR “COVID-19 Pandemics” OR (Pandemic AND “COVID-19”) OR “Coronavirus Disease 2019 Virus” OR “2019 Novel Coronavirus” OR “2019 Novel Coronaviruses” OR (Coronavirus AND “2019 Novel”) OR (“Novel Coronavirus” AND 2019) OR “Wuhan Seafood Market Pneumonia Virus” OR “SARS-CoV-2 Virus” OR “SARS CoV 2 Virus” OR “SARS-CoV-2 Viruses” OR (Virus AND “SARS-CoV-2”) OR “2019-nCoV” OR “COVID-19 Virus” OR “COVID 19 Virus” OR “COVID-19 Viruses” OR (Virus AND “COVID-19”) OR “Wuhan Coronavirus” OR (Coronavirus AND Wuhan) OR “SARS Coronavirus 2” OR (“Coronavirus 2” AND SARS) OR “Severe Acute Respiratory Syndrome Coronavirus 2”) | 655 |
| Google Scholar | Allintitle:violence covid | 399 |
